# Fatal Blastoid Variant Mantle Cell Lymphoma in a Patient with Sjögren's Syndrome

**DOI:** 10.1155/2013/831708

**Published:** 2013-04-23

**Authors:** Fabio Bonilla-Abadía, Manuel A. Pérez, Evelyn Muñoz-Buitrón, Joaquín D. Rosales, Carlos A. Cañas, Gabriel J. Tobón

**Affiliations:** ^1^Rheumatology Unit, Fundación Valle del Lili, ICESI University, Cali, Colombia; ^2^Internal Medicine Unit, Fundación Valle del Lili, CES University, Cali, Colombia; ^3^Investigative Unit, Fundación Valle del Lili, Cali, Colombia; ^4^Hematology Unit, Fundación Valle del Lili, ICESI University, Cali, Colombia

## Abstract

Primary Sjögren's syndrome (pSS) is an autoimmune disorder of the exocrine glands presenting with progressive ocular and oral dryness, parotid gland enlargement, and often with extraglandular manifestations. In this group of patients the risk of development of non-Hodgkin's lymphoma is 16-fold compared to healthy population, mainly of the MALT lymphoma type. This case reports a 52-year-old woman with pSS developing a progressively growing mass at face and neck compatible with blastoid variant mantle cell lymphoma and a fatal outcome.

## 1. Introduction

Primary Sjögren's syndrome (pSS) is an autoimmune disease characterized by lymphocytic infiltration of exocrine glands together with polyclonal B-cell activation [1]. Patients have an increased risk of up to 6% per year for developing non-Hodgkin's (NHL) B-cell lymphomas, including mucosa-associated lymphoid tissue (MALT) lymphomas in 70% of cases, preferentially extranodal marginal zone (MZ), located mainly in the major salivary glands [2]. This complication is associated with excess in the overall mortality rate in pSS patients. The association between pSS and lymphoma has been recognized since 1964 [3]; thus, attention is drawn to regular control of patients with this disease. Several clinical and serological markers have been reported to predict the development of NHL in pSS in different series. Among these parameters, patients with pSS and splenomegaly, persistent enlargement of parotid glands, lymphadenopathy, palpable purpura, cryoglobulinemia, low levels of C4, neutropenia, or lymphocytopenia have more than 5-fold increased risk of NHL compared to patients without risk factors.

This case reports a 52-year-old woman with pSS developing a progressively growing mass at face and neck compatible with blastoid variant mantle cell lymphoma and a fatal outcome.

## 2. Case Report

**Figure 1:**
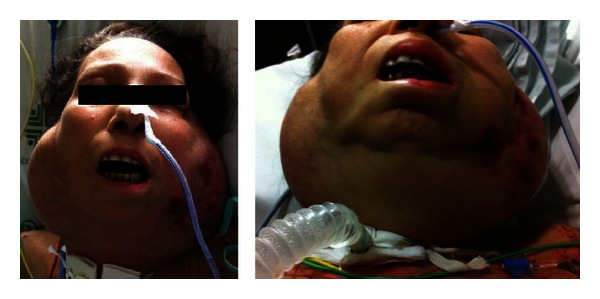
Bilateral increase in size of parotid glands.

**Figure 2:**
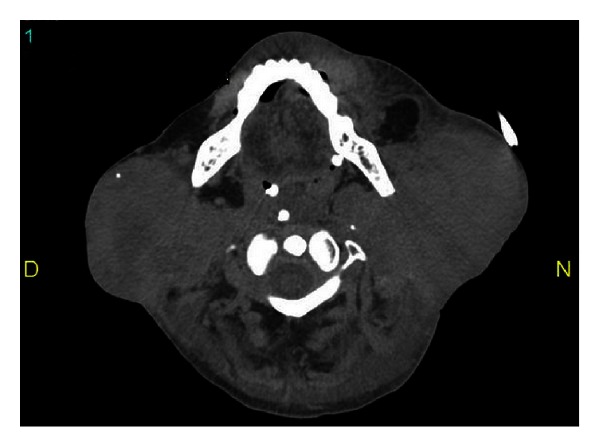
Neck computed tomography shows lymph node clusters in the neck associated with bilateral increase in size of parotid tissue.

**Figure 3:**
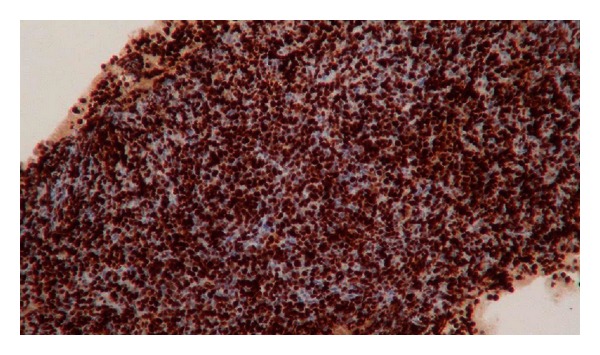
Cells expressing cyclin D1 in immunohistochemical study compatible with blastoid variant mantle cell lymphoma in a patient with primary Sjögren's syndrome.

A 52-year-old woman with a previous history of eight years of pSS (sicca syndrome, positive antinuclear antibodies, rheumatoid factor, and anti-Ro) was admitted to our hospital with the presence of a progressively growing mass on face and neck leading to swallowing and breathing difficulty. She reported chronic inflammatory arthralgias, xerostomia, and xerophthalmia. At physical examination on admission xerostomia and bilateral increase in size of parotid with dysphagia were observed (Figure 1). Cardiopulmonary, neurological, and osteoarticular systems were normal. On the biological analysis, low C4 and low C3 (65 mg/dL) levels were evidenced. High lactic dehydrogenase 777 U/L was also documented. Total leukocyte count was normal (6140 cells/mm^3^), but lymphopenia and thrombocytopenia were observed (platelets 75.000/mm^3^). Anti-HBs and anti-HCV antibodies were negative. Autoimmunity tests revealed a rheumatoid factor of 265 and direct Coombs test results was positive. Anti-cardiolipins antibodies were negative. Protein electrophoresis showed no gammaglobulins peak. A brain and neck computed tomography (CT) was made observing sphenoidal acute sinusitis and lymph node clusters in the neck associated with bilateral increase in size of parotid tissue (Figure 2). A chest CT was performed showing diffuse lymphadenopathy and necrotic areas in supra- and infraclavicular cervical node chains with compressive effect on the venous structures and upper airway. Abdominal CT showed para-aortic, mesenteric, and bilateral hypogastric lymphadenopathy in external iliac and inguinal chains. She was managed in intensive care unit (ICU) with ventilator support and dexamethasone initially. Tracheostomy and gastrostomy were required. A fine-needle aspiration of the left parotid gland was performed and immunohistochemistry study showed cells expressing cyclin D1 compatible with blastoid variant mantle cell lymphoma (Figure 3). Immunochemotherapy onset was indicated. No satisfactory response was evident; the patient developed septic shock and died despite of wide antibiotic treatment and support in the ICU.

## 3. Discussion

Biologic abnormalities associated with B lymphocytes in pSS are manifested as rheumatoid factor, hypergammaglobulinemia, and anti-SSA and anti-SSB antibodies, together with an increased risk of NHL up to 5% of the patients [4, 5]. Usually several years elapsed between pSS diagnosis and lymphoma (7.5–14 years). This complication is associated with excess in the overall mortality rate in pSS patients. Various histological subtypes of lymphoma have been described, including follicular lymphoma, diffuse large B-cell lymphoma (DLBCL) (30% of cases), and especially MALT lymphoma (70%), preferentially extranodal MZ, located mainly in the major salivary glands (SG) (80% of cases). Lymphomas of the SG are rare and account for 4.7% of lymphomas at all sites [6]. An NHL of an SG may appear as a painless, progressively enlarging mass [7, 8]. SG lymphoma should be considered in the differential diagnosis of cystic or bilateral SG lesions. In this case, we report a rare blastoid variant of mantle cell lymphoma associated with pSS. To our knowledge this is the first reported case of this variant of mantle cell lymphoma in a patient with pSS.
